# Barriers and facilitators of child-feeding practice in a small sample of individuals from Gozamin District, Northwest of Ethiopia: a qualitative study

**DOI:** 10.1186/s40795-018-0233-z

**Published:** 2018-05-23

**Authors:** Nakachew Mekonnen, Shifera Asfaw, Abebe Mamo, Yared Mulu, Netsanet Fentahun

**Affiliations:** 1 Department of Public Health, Debre Markos University, Debre Markos, Ethiopia; 2 Department of Health Education and Behavioral Sciences, Jimma University, Jimma, Ethiopia; 3 School of Public Health, College of Health Sciences, Bahir Dar University, Bahir Dar, Ethiopia

**Keywords:** Child feeding, Exploring practices, Qualitative study, Amhara region, Ethiopia

## Abstract

**Background:**

The first 1000 days is “window of opportunity” for nutrition and vital for physical growth, brain development and the immune system. None of previous studies explored qualitatively child-feeding practices in the developing countries like Ethiopia. The aim of the study was to explore barriers and facilitators of child-feeding practices in Gozamin District, Northwest Ethiopia.

**Methods:**

A qualitative study was conducted by using 12 in-depth interviews and 4 Focus Group Discussions (*n* = 32) from Feb. 15/2016 – March 10/ 2016 in eight Kebeles. Purposive sampling technique was used to recruit the participants. The quality of the research findings was checked by using credibility, dependability, transferability and conformability. Data were analyzed using qualitative data analysis software package Atlas ti-7.

**Results:**

Early initiation of breast-feeding and complementary feeding, exclusive breast-feeding, minimum meal frequency and minimum dietary diversity were the emerged theme in the study. Breastfeeding up to 2 years and above and timely initiation of a complementary feeding were commonly good practice in the area. Dietary diversity, discarding colostrums, pre-lacteal feeding like butter and bottle-feeding were the commonly harmful practices in the area. The most frequently mentioned barriers of child feeding were socio-cultural influences, traditional community practices, workload and poverty.

**Conclusions:**

Most of the children were suffered from harmful child feeding practices such as pre-lacteal feeding, discarding colostrums and bottle-feeding. Child dietary diversity and complementary food preparation were the major problem in the study area. Therefore, supports on complementary food preparation and diversity food should be given more attention to enhance child-feeding practice in rural Ethiopia.

## Background

The first 1000 days is window of opportunity for physical growth, brain development and the immune system [1]. Child feeding practices include both breastfeeding and complementary feeding practices [2]. Sub-optimal breastfeeding in the first 6 months of life, results in 1.4 million deaths and 10% of the disease burden in children younger than 5 years [3–5]. Early initiation of breastfeeding result in better establishment of exclusive breastfeeding practices and continued breastfeeding [6–10].

Poor breastfeeding and complementary feeding practices have been widely documented in the developing countries. In Ethiopia, Mothers give herbal extracts to their children, discarded colostrums, and introduced complementary feeding as early as 3 months [11, 12]. The early introduction of complementary feeds before the age of 6 months increased risk of infections and further contributes to malnutrition [13]. Optimal complementary feeding involves factors such as the quantity and quality of food, frequency and timeliness of feeding, and feeding during/after illnesses [14–16].

The Federal Ministry of Health of Ethiopia has tried to enhance the practice of optimal child feeding practice by developing training manuals and implementation guidelines; and incorporated it to the primary health care in line with the health extension program. Quantitative studies were conducted in the Amhara region related to child feeding practice. But, there is no qualitative study which helps to deep understanding of the child feeding practice phenomena in the Amhara region. Therefore, this study aimed to explore barriers and facilitators of child-feeding practices in Gozamin District, Northwest Ethiopia child-feeding practices in Gozamin District, which is one of the rural part of Amhara regional state, Northwest, Ethiopia.

## Methods

### Study area and period

The study was conducted in Gozamin District, Amhara Regional State Northwest Ethiopia. The district is found near Debere Markos town which is located in Northwest of Ethiopia which is 300 Km away from Addis Ababa capital city of Ethiopia. The total population of the district was projected to 153,151 for 2016 and 13.4% of them are under 5 year children and estimated number of under 2 years old children was 5.2%. It comprises a total of 26 Kebeles (the smallest unit of administration), 26-health post and six health centers. Most of the communities are farmers and earns their income from agriculture. The main products of the district are cereals crops like wheat, maize, barley, teff, etc. The feeding habits of the community are feed together with one dish in the family. The usual foods of the community are Injera from wheat and teff with Shiro wot. This study conducted from February 15/2016 – March 10/2016.

### Study design

A qualitative study was conducted to explore barriers and facilitators of child-feeding practices in Gozamin District, Northwest Ethiopia.

### Study population

Selected mothers/caretakers who had a child under age of 2 years, health extension workers, grandmothers, and community leaders who are reside in the kebeles for at least 6 months in the District were included in the study. To recruit mothers who had children less than 2 years of age; the immunization records of the health post in the kebeles was used.

### Sample size and sampling technique

Among 26 rural kebeles, eight were selected by using purposive random sampling technique. A small cohort of study participants (40 females and 4 males) were participated in this study. Twelve in-depth interviews and four focus group discussions (*n* = 32) were conducted. The in-depth interview was conducted among 4 health extension workers, 4 grandmothers, 2 grandfathers and 2 kebele leaders. Purposive-criterion sampling technique was used to recruited in-depth interview participants. The in-depth interviews included different actors in the community which had diverse influence on child feeding practice. Some of the basic criteria which were employed to recruit participants were; their profession (health extension workers), the significant important in the social system (grandfather and grandmothers and their leadership position in the community (kebele leaders).

The reason why we used triangulations multiple data sources and diversify the study participants in terms of age, residence, and position for deeper understanding of the child practice were to assure data quality. Four focus group discussions were conducted among mothers/caregiver who had children under age of 2 years. Mothers/caretakers who had registered children less than 2 years of age from the immunization record book were included in the study.

### Data collection technique and procedures

Data were collected through in-depth interview and focus group discussions by using interview and focus group discussion guides. The participants were engaged with participants posing questions in a neutral manner, listening attentively and asking follow up and probing questions after participants’ response were done. The guides consist of the socio-demographic characteristics like age, position/status of the individual, sex, level of education, religion, child feeding practices, barriers and facilitators of child feeding practices.

The in-depth interviews were performed by the principal investigator with one in-depth interview per a day in order to accommodate participants’ availability and immediate transcription. Each focus group discussion and in depth- interview was held in the morning in the area where the place and time was convenient for the participants. All focus group discussions conducted by a team of three, including a post basic public health officer as a moderator, masters in general public health professional as a facilitator and principal investigator as a note taker, and an observer. During the interview and focus group discussions, field note was taken. Data were recorded via digital voice recorder and each in–depth interview and focus group discussions were taking of 45–90 min.

### Data processing and analysis

The data were transcribed and analyzed immediately after each interview and focus group discussion. The field notes were rewrite again accordingly and the records were listened repeatedly to understand the concepts of each participant’s response. To ensure rigor and check the quality of the data analysis codes/pseudonyms were allocated to the participants to maintain anonymity, each transcription and record were assigned an identification number. To test and verify coding validity, transcripts were coded separately by the principal investigator and another qualitative researcher who is working in Jimma University.

All interviews and focus group discussions were transcribed verbatim directly from audios into Amharic transcripts initially by the principal investigator. The Amharic transcripts were translated in to English by the principal investigator and other Public health professional to keep the consistency of the data and to minimize researcher’s bias. Data were analyzed inductively; using qualitative data analysis software package Atlas ti-7. Each transcript was screened quoted and coded by the two coders. These codes in turn were grouped into major families and then into themes representing reported child-feeding practices. For each interviewee and focus group discussion, identified themes were outlined in a chronological order Finally, the most frequently cited sequence of child feeding practices were identified for themes formation. In presenting the data, relevant verbatim quotes were reported to aid the interpretation of the data in each themes and categories.

### Data quality assurance

A. Credibility

In order to maintain credibility, the research findings both in-depth interview and focus group discussion guidelines were evaluated by the professionals, before the data collection. The two individuals who were participated on the focus group discussions, orientation about the purpose of the focus group discussion and responsibility was given before the focus group discussions takes places to avoid unnecessary interruption and keep the rights of the participants. Triangulations were made by using multiple data sources and diversify the study participants in terms of age, residence, and position for deeper understanding of the child practice.

B. Transferability

To maintain transferability of the finding, appropriate probes were used to obtain detail information on responses. Detailed field notes and digital audio recordings were done for all interviews and focus group discussion before and during analysis (thick description).

C. Dependability

To maintain dependability of the research process member checking was made by returned back the preliminary findings to the participants to correct errors and challenge what were perceived as wrong interpretations. Prolonged engagement was made to address individuals at different rang trust was built with participants.

D. Conformability

To insure Conformability of the research process persistent observations were observed on their body movements; facial expression, eye gaze, tone of speech each and every thing was recorded at the time of interview and focus group discussions. Peer debriefing made by exposed the preliminary findings to Master of Public Health (MPH) professional at Debere Markos University to exploring aspect of the inquiry that might remain only implicit within the inquirer’s mind. Further, throughout the study reflectivity and bracketing methods were employed in order to minimize respondent bias and the risk of reactivity whereby participants could withhold information due to the presence of a researcher “Bracketing out” can be used for holding back researchers’ preconceived ideas about the issue under study, and this was done by forwarding open-ended questions arranged to follow the cues from participants.

## Results

### Characteristics of respondent

A total of 44 individuals were participated in the study for both 4 Focused Group Discussion (FGDs) and 12 In-depth interviews. A mean age of the study participants was 32.8(SD ±10.09) years, ranging from 19 to 55 years (Table 1).

**Table 1:** Showed distribution of the socio-demographic characteristics of participants, Gozamin District, Northwest Ethiopia, 2016

		Frequency	Percent
Sex	Female	40	90.9
	Male	4	9.1
Marital Status	Married	38	86.4
	Single	3	6.8
	Others^a^	3	6.8
Educational Level	Illiterate	30	68.2
	Primary education	8	18.2
	Secondary and above	6	13.6
Role/Position	House wife	32	72.7
	Health Extension Workers	4	9.1
	Grand mothers	4	9.1
	Kebele and community leaders	4	9.1
	Total	44	100.0

^a^Others: Divorced, Widowed, and Separated

### Child feeding practices

A practiced performed by the mothers/caretakers should be as per world health organization recommendations: exclusively breastfed for the first 6 months, timely introduction of complementary feeding (6–8 months), appropriate and safe complementary foods from 6 months up to 2 years along with breastfeeding (Table 2).

**Table 2:** Summary of the themes and categories of child feeding in Gozamin District, Northwest Ethiopia, 2016

Themes	Categories
Breast feeding practices	• Early initiation of breast feeding
	• Exclusive breast feeding
	• Duration of breast feeding
	• Frequency of breast feeding
Complementary feeding practices	• Timely initiation of complementary feeding
	• Diversity and Preparation complementary feeding
	• Frequency of complementary feeding
Facilitating factors of child feeding practices	• Socio-cultural influences
	• Community practices
Barrier of child feeding practices	• Workload
	• Beliefs
	• Economy

***Theme - 1: Breast feeding practices***

***Category - 1: Initiation and exclusive breast-feeding***

Most mothers/ caretakers reported that they initiate breast-feeding immediately after birth; this happens when a mother give birth at the health institution as a result of an opportunity to get information on childcare.

*“I was giving a birth at the health institution; ... I was started to feed immediately after birth. I was fed the first milk of the breast too and I am feeding my child properly”.*

**(Mother, age from 20 to 25 years)**

Some of the mothers/caretaker was given butter immediately after birth for child to initiation a child for feeding (“Menakekia in Amharic”). The reason why mother gave butter child has good voice in the later life, to make the child patience, and to clean the intestine; as well to smooth the esophagus of the child for further feeding.

Most mothers/caretakers confirmed that the introduction of water, milk, butter, and other foods/liquids before 6 months is not a usual practice. Many participants are aware of the recommendation to breastfeed exclusively

*“…a child whose age less than six months, at any time the child will be feed breast milk only, no water or anything else is given for them ”.* **(Female, age from 35-40 years)**

Some mothers/caretakers did not feed colostrums for their child. They believed that “colostrums” is the cause for diarrhea and will be made the child too much tiny, unhealthy, and weaker in the later life of the child.

*“Immediately after birth; the mothers will be discharged the first breast milk … Colostrums will cause a wound on the throat of the child if it feeds and it makes the child very thin and causes diarrhea*”. **(Kebele leader, age from 45-50 years)**

***Category-2: Duration and frequency of breast-feeding***

Most mothers/caretakers reported that continued breastfeeding until the child is 2 or 3 years old. Many of the women will be stopped breastfeeding either when the child is old enough to eat on his or her own or when they/mothers/become pregnant.

*“Currently; the mothers/caregivers breast feed their child up to the age of three years. But, previously it was difficult to feed a child till three years of age. These were because of frequent pregnancy due to lack of contraceptive methods for child spacing”.* **(Grandmother, age range 50-55 years)**

All most all mothers/ caretakers reported that breastfeed frequently depend on child demand. But, it was difficult for all participants to quantify how many times they breastfeed during the day and night.

*“I am feeding the child breast milk ten times per day. Even, more than ten times”.* **(Mother, age from 25-30 years)**

**Theme-2: Complementary foods feeding practices**

***Category - 1: Initiation and frequency***

Majority of mothers/ caretakers reported that complementary feeding started at age of 6 months. But, some mothers/caretaker stated that mothers started an additional food before the recommended time nearly around 3 months. The primary reasons given for introducing food early were mothers perceived that when they start field works, the quantity of the breast milk becomes decreased and that additional foods are needed to avoid hunger and ensure good growth and strength.

And some of mothers/caretakers had a tendency to introduce foods beyond 6 months.

*“…the esophagus is not mature enough to allow food passage, and they don't have a tooth. After 9 months onwards, the child started to bring everything they got to their mouth. If not doing this and if s/he didn't erupt a tooth no one will start to feed a complementary food for a child”.* **(Kebele leader, age from 45-50 years)**

Children are being fed according to the recommended minimum frequencies for each age group, at least three, four, and five times for children age 6–8, 9–11, and 12–24 months respectively. Mothers/caretakers reported that they feed food to their children frequently, ranging from two to four times per a day.

***Category-2: Diversity and Preparation***

Almost all mothers/caretakers reported that soup/ “*Atimit in Amharic”*/and porridge prepared from at least two types of cereals. The predominant cereals for the preparation of the complementary food were “Dabo teff”, Keyie teff and other cereal crop cultivated by the community.

*“…mothers prepare soup* (*Atimit in Amharic*) *from Bread, Dabo teff and Keyie teff. However, Injera with watt will not be given for child at until one year”.* **(Mother, age range 40-45 years)**

Nearly all mothers/caretakers explained that there had no practice to buy commercially prepared food products to feed their child in the area due to lack of accessible and expensive.

*“…No practice buys commercially prepared food products in the community to the feed the children. The community prepared their own food items which are available in their house. No one expenses money to feed a child”.* **(Kebele leader, age from 40-45 years)**

**Theme-3: Facilitating factors of child feeding practices**

***Category-1: Socio-cultural influences***

Mothers/caretakers stated that at the time of birth there is a special ceremony which is done for the one who gave birth. The neighbors prepare special food teff flour with butter until 45 days. At the time of the visit all visitors give advice to feed her breast frequently and to keep the cleanness of the child each day, especially if the mother gave the first birth; including the feeding patterns, styles etc. These are opportunities for the mother to practice appropriate feeding practices.

*“Not only the mother, but also the neighbor … For myself, my wife was giving birth; immediately after birth, sheep was slaughtered for festive for who gave birth. Those individuals who have no potential, they called the neighbors and prepare porridge for festive. Then after the entire neighbor and the family gave care for the one who gave birth; intern the mother for the child”.* **(Opinion Leader, age range from 50-55-years)**

***Category -2: Community practices***

All mothers/caretakers stated that they are engaged in one to five networking; these groups contain at least one model family. The networking used to share experience. All attend a session on counseling in different health issues including child health at least once per 2 weeks. The model families create a competition to their success. At all these make an opportunity for most mothers to experience child-feeding practices.

*“We have immunization sessions every month. When the sessions are implemented there is a show how to prepare in rich food for a child … Mothers tried to prepare in rich foods for their child; and others prepare with which they have at house level*”. **(Health Extension Worker, age range from 25-30 years)**

Most mothers/caretakers stated that Health Extension Workers tried to show how mothers prepare diversified food from the food items which the families have.

*“Most of the time mothers prepared the complementary food to their children mixing up together from “Dabo teff”/Keyie teff/, wheat, barley and maize, beans and peens. To guide as expertise; we usually interfere in the preparation of the complementary food as the mothers prepare for their children”. (* **Health Extension Worker, age range from 25-30 years)**

Key informants stated that mothers/family in the Kebele has a family guide book which contains messages on breast feeding, complementary food preparation, personal hygiene, Insecticide Treated Net utilization, etc.

*“Most mothers are practicing feeding a child because all household in this community have family guide book and we teach them through for those mothers who cannot read and write the book also has pictorial presentation easily to understand it”.(* **Health Extension Worker, age range from 25-30 years)**

**Theme - 4: Barrier of child feeding practices**.

**Category-1: Workload**

All mothers/caretakers stated that we were not properly feed our children due to different workload from outside and inside at home.

*“… at some time the mothers loaded with work; at this period, they breast feed their child at the time when the child cries. But, if the mothers are not busy they feed breast milk more than the standard what it was recommended”.(* **Health Extension Worker, age range from 25-30 years)**

**Category -2: Beliefs**

Mothers/caretakers stated that the belief on child feeding practice in the study area is a problem. Despite, the efforts made to show and demonstrate diversified food preparation during immunization sessions and house-to-house visits by Health Extension Workers; the community becomes resistance what it is said and has had not practice it. Flesh foods, such as poultry, meat, and fish are not commonly given to young children in their communities. The reasons were children are too young to chew and digest the foods.

*“In this community, foods which are not eating by the child are meats. Especially; goat’s meat is not given and eaten by most individuals; these is because, there is a belief which cause teeth problem”.* **(Mother, age range from 20-24 years)**

Mother’s practices bottle-feeding by assuming, the bottle-feeding is clean, keep covered, and the food is not contaminated easily. They believed that a cup is not hygienic and children are not able to drink from a cup. In addition, open utensils were prone to contamination and the fluid might be poured out at the time of feeding and are not convenient to handle and feed the child.

*“I gave fluids like* soup/ “*Atimit in Amharic”*/*and milk with a bottle with cover. The bottle decreases the risk of contamination, convenient to handle and feed the child”.* **(Mother, age range from 20-24 years)**

**Category - 3: Poverty**

Most of the Health Extension Workers said that the children in their communities are not getting a variety of foods for a number of reasons. Families do not have the ability to buy various types of fruits, vegetables and cereals, so families just prepare food from what is available at hand.

*“The health professional told us many times to prepare diversified complementary food with meat, egg, cabbage … etc. However, we are not doing so. Some mothers/caregivers can't access it; on the other hand, it is a problem of awareness and negligence. Nothing else for the poor practice for me and the community too”.* **(Mother, age range from 20-24 years)**

## Discussion

The present study has primarily aimed to explore the child feeding practices (children below 24 months). The study has shown that almost all women have ever breast fed their children. Breastfeeding is one of the best ways to ensure child health and survival [3]. Early initiation of breastfeeding provides benefits for both the infant and mother [10]*.* In this study women initiated breast-feeding early. The finding is similar with other studies reported in Ethiopia [17]. Breast-feeding is a norm in most parts of Ethiopia. Especially in the study area if mothers are not breast feed they will be criticized by the community.

Once women start breast-feeding their children, consistent with evidence that breastfeeding is almost universal in Ethiopia with over 98% of children ever breastfed at some point [12, 18] majority of participants reported consistently continuing the practice for longer period [19]. In this study, most participants continued breast-feeding until age 2 and above. This practice is promoted and recommended by WHO [2] and similar with the other studies in the region [12]. Initiation of breastfeeding within an hour and prelacteal feeding (like Butter) are the common problem in the study area. Culturally, the community consider butter as the food which cleans the stomach and smooth the esophagus of the child.

Colostrum avoidance is practiced in the study community and these align with the study in Northern Ethiopia [20]. Culturally; colostrums considered as impure and the cause for a child to become thin, unhealthy, and weaker in the later life of the child. Complementary feeding is the process starting when breast milk alone is no longer sufficient to meet the nutritional requirements of an infant [10]. Timely introduction of adequate, safe, and diversified complementary food is necessary at 6–8 months to meet energy and nutrient requirements [17]. The timely initiation of complementary foods and dietary diversity are the common problem in the study area. The finding aligns with a study done in Tigray and Southern Nation Nationality and People of Region [21].

Even though the mothers attend how to prepare diversified food, they did not practice it. Cultural beliefs, workload and poverty are main barriers for optimal child feeding practices in the study area. In contrast, there are opportunities to enhance child-feeding practices through one to five networking and model families. There is a demonstration regarding complementary food preparation in each locality. A study conducted in Gambia, revealed that; the elders in the community also give support at family level [22]. Using a bottle for feeding a child, a practice that is discouraged increases the child’s risk of illness and reduces the child’s interest in breastfeeding, with consequent potential decline in milk production [2]. In this finding, bottle-feeding is practiced in the study community.

## Conclusion

Children were suffered from harmful child feeding practices such as pre-lacteal feeding, discarding colostrums and bottle-feeding. Child dietary diversity and complementary food preparation were the major problem in the study area. Therefore, supports on complementary food preparation and diversity food should be given more attention to enhance child-feeding practice in rural Ethiopia.

## Abbreviations

FGD: Focused Group Discussion
MPH: Master of Public Health
SD: Standard Deviation
WHO: World Health Organization
